# Protection against *Mycobacterium tuberculosis* Infection Offered by a New Multistage Subunit Vaccine Correlates with Increased Number of IFN-γ^+^IL-2^+^ CD4^+^ and IFN-γ^+^ CD8^+^ T Cells

**DOI:** 10.1371/journal.pone.0122560

**Published:** 2015-03-30

**Authors:** Xiaochun Wang, Jingyan Zhang, Jinping Liang, Ying Zhang, Xindong Teng, Xuefeng Yuan, Xionglin Fan

**Affiliations:** 1 Department of Pathogen Biology, School of Basic Medicine, Huazhong University of Science & Technology, Wuhan, P.R. China; 2 Department of Molecular Microbiology and Immunology, Bloomberg School of Public Health, Johns Hopkins University, Baltimore, Maryland, United States of America; Federal University of São Paulo, BRAZIL

## Abstract

Protein subunit vaccines present a compelling new area of research for control of tuberculosis (TB). Based on the interaction between *Mycobacterium tuberculosis* and its host, five stage-specific antigens of *M*. *tuberculosis* that participate in TB pathogenesis—Rv1813, Rv2660c, Ag85B, Rv2623, and HspX—were selected. These antigens were verified to be recognized by T cells from a total of 42 whole blood samples obtained from active TB patients, patients with latent TB infections (LTBIs), and healthy control donors. The multistage polyprotein A1D4 was developed using the selected five antigens as a potentially more effective novel subunit vaccine. The immunogenicity and protective efficacy of A1D4 emulsified in the adjuvant MTO [monophosphoryl lipid A (MPL), trehalose-6,6′-dibehenate (TDB), components of MF59] was compared with Bacillus Calmette-Guerin (BCG) in C57BL/6 mice. Our results demonstrated that A1D4/MTO could provide more significant protection against *M*. *tuberculosis* infection than the PBS control or MTO adjuvant alone judging from the A1D4-specific Th1-type immune response; however, its efficacy was inferior to BCG as demonstrated by the bacterial load in the lung and spleen, and by the pathological changes in the lung. Antigen-specific single IL-2-secreting cells and different combinations with IL-2-secreting CD4^+^ T cells were beneficial and correlated with BCG vaccine-induced protection against TB. Antigen-specific IFN-γ^+^IL-2^+^ CD4^+^ T cells were the only effective biomarker significantly induced by A1D4/MTO. Among all groups, A1D4/MTO immunization also conferred the highest number of antigen-specific single IFN-γ^+^ and IFN-γ^+^TNF-α^+^ CD4^+^ T cells, which might be related to the antigen load *in vivo*, and single IFN-γ^+^ CD8^+^ T cells by mimicking the immune patterns of LTBIs or curable TB patients. Our strategy seems promising for the development of a TB vaccine based on multistage antigens, and subunit antigen A1D4 suspended in MTO adjuvant warrants preclinical evaluation in animal models of latent infection and may boost BCG vaccination.

## Introduction

Protein subunit vaccines, consisting of defined immunodominant antigens of *Mycobacterium tuberculosis (M*. *tuberculosis)*, are sufficiently safe as a stand-alone prophylactic vaccine in a population where Bacillus Calmette-Guerin (BCG) immunization is not recommended, or as a boost to the currently used BCG vaccine. Half of twelve tuberculosis (TB) vaccine candidates that are currently entering clinical trials belong to the protein subunit category, demonstrating the important role of this type of vaccine in the next generation of TB vaccination [1, 2]. Nevertheless, the selection of protective antigens has mainly focused on proteins released from the growing *M*. *tuberculosis* bacteria into the culture medium *in vitro* [3, 4], and only a few antigens have been identified so far as promising targets for TB subunit vaccines [1, 2]. Suitable antigens are mainly selected on the basis of antigenicity, immunogenicity, and anti-infection protection in various animal models [5]. These methods may face inherent limitations to antigen screening due to the complex pathogenesis of *M*. *tuberculosis* and its interaction with the host as demonstrated in a recent review [6], thereby significantly hindering the development of effective subunit vaccines.

The pathogenesis of *M*. *tuberculosis* progresses successively through primary infection, latency, and reactivation. During these infection stages, the metabolism and gene transcriptional profiles of *M*. *tuberculosis* show significant differences and stage-specific features *in vivo* [7–10]. Once inhaled into the pulmonary alveoli, *M*. *tuberculosis* can reside and even replicate in the macrophages, thereby evading the innate immune response against infection of the host through immunosuppression [11, 12] and immune evasion [13, 14]. In a mouse model of primary infection, *M*. *tuberculosis* grew exponentially over 2 to 3 weeks following infection *in vivo* [15] and mainly secreted antigens such as the Ag85 complex [16] and the ESX protein family [17] when grown in liquid medium *in vitro*. Subsequently, T cells, especially CD4^+^ Th1-type cells that were sensitized in the local lymph node and homing the infected lesion via the pulmonary circulation, can secrete Th1-typed cytokines such as IFN-γ, which activates macrophage phagocytosis, thereby killing intracellular bacteria, and TNF-α, which attracts more infiltrating macrophages and lymphocytes and promotes granuloma formation at the site of infection, ultimately leading to latent TB infection (LTBI). During this chronic phase, the metabolic status of *M*. *tuberculosis* aggregating in the granuloma transfers from growing replication to dormancy *in vivo* with limited antigen presentation [18]. In addition, several mycobacterial genes involved in metabolism conversion such as *Rv*2660c [19], and 48 *DosR*-regulon genes [10] including *HspX* [20], *Rv1813* [21], and *Rv2623* [22], were significantly up-regulated. Dormant *M*. *tuberculosis* persisting in the host can reactivate under conditions of suppressed immunity of the host such as co-infection with HIV, thereby resulting in the formation of endogenous infection and the development of active pulmonary TB in the adult. Although the BCG vaccine confers the CD4^+^ Th1 response and protects effectively against serious forms of TB during childhood, the protective efficacy gradually decreases over time and does not protect against pulmonary TB in adults or against reactivation from LTBI [23, 24]. This might be attributed to low or masked IFN-γ responses to latency antigens induced by BCG vaccination [21, 22, 25], although the genes encoding latency antigens are highly homologous between BCG and *M*. *tuberculosis*. Promising targets for TB vaccines were previously focused on antigens from the Ag85 complex and the ESX protein family secreted by *M*. *tuberculosis* during primary infection. More recently, several latency antigens such as Rv2660c [19], Rv1733 [26], Rv1813 [21], and HspX [27] were used to construct fusion multistage vaccine candidates such as H56 [19, 28] and ID93 [29], which were shown to provide protection against primary and latent infection and even MDR-TB in animal vaccination models. These studies partially confirm the vaccine potential of stage-specific antigens expressed by *M*. *tuberculosis*.

The aim of the present study was to develop a more effective multistage subunit vaccine. Antigens expressed during different stages of *M*. *tuberculosis* were screened and confirmed on the basis of immune recognition by T cells from TB patients and LTBIs. A multistage polyprotein based on five selected antigens was constructed and its immunogenicity and protective efficacy was compared with BCG in a C57BL/6 mouse model.

## Materials and Methods

### Prokaryotic expression of recombinant proteins in *E*. *coli*

The coding sequence of the A1D4 fusion protein consisting of antigens Rv1813, Rv2660c, Ag85B, Rv2623, and HspX, was PCR-amplified using primers specific for the recombinant plasmid pDC316-A1D4, which was commercially synthesized by Life Invitrogen (Shanghai, China). Genes encoding the antigens Rv1813, Rv2660c, and Rv2623 were amplified by PCR with their respective primers from the genomic DNA of *M*. *tuberculosis* H37Rv (S1 Table). All PCR products were digested with *Nde*I and *Xho*I restriction enzymes and then inserted into the corresponding sites of the prokaryotic expression vectors pET-30b or pET28a, respectively. Recombinant prokaryotic expression plasmids were verified by enzyme digestion, and the inserted DNA fragments were confirmed by DNA sequencing. The recombinant plasmids were transformed into the *E*. *coli* strain BL21 (DE3), and the expression of the target proteins was induced by incubation with isopropyl thio-β-D-galactoside (IPTG) at a final concentration of 1 mM for 4 h. Recombinant proteins were purified using NTA-metal ion affinity chromatography according to the manufacturer’s instructions (GE Healthcare, NJ, USA). Successful expression and purification was monitored by 15% SDS-PAGE. The specificity of recombinant proteins was confirmed by western blotting with an anti-His 6 mouse monoclonal antibody (mAb, diluted 1/2000; Tiangen, Beijing, China) or anti-A1D4 protein mouse serum as the primary antibody and peroxidase-conjugated goat anti-mouse IgG (diluted 1/5000; Proteintech Biotech, Wuhan, China) as the secondary antibody. The protein bands on immunoblots were visualized using enhanced chemiluminescence (ECL) technology (Tiangen, China). Recombinant proteins were dialyzed against sterilized PBS with a urea concentration gradient (from 8 M to 0 M) for 48 h at 6 h intervals. Proteins were lyophilized, diluted in phosphate-buffered saline (PBS) using pyrogen-free reagents, aliquoted, and stored at -20°C. Residual endotoxin contamination was verified to be below 0.1 EU/mL of protein. The protein concentration was determined using a BCA Protein Assay Kit (Beyotime, Shanghai, China). The recombinant proteins Ag85B [30], HspX [30], and rCM (CFP21-MPT64) [31] were prepared as previously described.

### Ethics statement

The study protocol was approved by the Ethics Committee of Tongji Medical College (Permit Number: 201201). Patients were enrolled from Xinxiang Institute of TB Prevention and Treatment (Xinxiang, Henan Province, China), and written informed consent was obtained from all subjects; for children the informed consent was provided by their parents. Animal experiments were performed in accordance with the guidelines of the Chinese Council on Animal Care. The research protocol was approved by the ABSL-3 Lab of Wuhan University (Wuhan, China) and the Committee on the Ethics of Animal Experiments of Wuhan University (Permit Number: S01312122Q). All surgery was performed under sodium pentobarbital anesthesia, and every effort was made to minimize suffering.

### Human clinical samples

All participants were recruited for screening based on the diagnostic standards listed in S2 Table Active TB patients were diagnosed by history, clinical symptoms, positive sputum smear microscopy, and aberrant lung X-ray. Healthy LTBIs had a history of household contact with sputum-positive TB patients in the preceding six months and a tuberculin skin test (TST) result of ≥5 mm, while clinical symptoms and aberrant lung X-ray were absent in these patients. In order to avoid any effects of BCG vaccination on the results, all TB infections were further screened by recombinant CFP21-MPT64-whole blood IFN-γ assay (rCM-WBIA) as rCM-WBIA-positive TB patients and LTBIs based on rCM-specific IFN-γ concentration with a cut-off point of 398.5 pg/mL [31]. Healthy donors were diagnosed with no history of prior *M*. *tuberculosis* contact or exposure, and negative results of both TST and rCM-WBIA. All subjects were seronegative for HIV infection.

### Whole blood IFN-γ assay based on rCM, A1D4, and subfractions of A1D4

Before treatment, 5 mL of fresh heparinized whole blood was collected from each donor, and 0.5 mL was seeded in each well of a sterile 24-well plate. rCM (20 μL), A1D4, and subfractions of A1D4 (10 μg/mL each) were used in each well for stimulation and incubated for 18 to 24 h at 37°C. PHA was used as a positive control at a final concentration of 20 μg/mL. Saline solution served as the negative control. Following stimulation, 200 μL of supernatant were collected from each well and stored at -20°C until further detection. The IFN-γ concentration was quantified using a commercial human IFN-γ assay kit according to the manufacturer’s instructions (Dakewe Biotech, Shenzhen, China). The antigen-specific concentration of IFN-γ in each sample was calculated by subtracting the value of the saline control from the value of the sample stimulated with the respective antigen. The detection limit of the ELISA was 4 pg/mL and the assay was set arbitrarily at 100 pg/mL for PHA-WBIA. The cut-off value for a positive response to each multistage antigen was set arbitrarily as 3X the mean of the negative control value of healthy controls. The results were analyzed as previously described [32].

### Animals and immunization protocol

Specific pathogen-free, 6- to 8-week-old female C57BL/6 mice were obtained from Wuhan University Center for Animal Experiment and were bred in separate cages on an animal feeding cabinet (VentiRack, CA, USA). The protein subunit vaccine contained 20 μg of A1D4 emulsified in the adjuvant MTO composed of MPL (50 μg/dose; Avanti Polar Lipids Inc., AL, USA), TDB (50 μg/dose; Avanti Polar Lipids Inc., USA), and components of MF59 including squalene oil (1%, v/v), Tween 80 (0.4%, v/v,), and Span 85 (1%, v/v, all purchased from Sigma-Aldrich, MO, USA). For this study, 0.2 mL of vaccine preparation was given to mice subcutaneously (s.c.) three times at 3-week intervals. BCG China was used as a positive control and inoculated s.c. once with 1.67 × 10^6^ CFU at the time of the first vaccination. PBS and adjuvant MTO alone were separately used as negative controls.

### Antigen-specific antibody titers

Three weeks after the last immunization, sera were collected from each mouse and A1D4 specific endpoint titers for IgG1, IgG2c, and IgG were detected by enzyme-linked immunosorbent assay (ELISA). Microtiter plates were precoated with 100 μL A1D4 protein (5 μg/mL) or BSA (irrelevant control) in carbonate/bicarbonate buffer (pH 9.6) overnight at 4°C and blocked with 1% BSA in PBST for 1 h at 37°C. Sera from mice treated with PBS were used as a negative control. Serial dilutions of serum samples were added, followed by addition of horseradish peroxidase-conjugated rabbit anti-mouse IgG (Ab6789, 1/5000), IgG1 (Ab97240, 1/10000), or IgG2c (Ab97255, 1/10000, all antibodies from Abcam, Cambridge, UK). Colors were developed with 3,3′,5,5′-tetramethylbenzidine (TMB) substrate and the enzymatic reaction was stopped with 1 N H_2_SO4. Plates were read at 450 nm using a microplate ELISA reader with the detection limit set at 0.05 at OD 450 nm. Antibody titers in each group were expressed as reciprocal end point titers by comparison with the PBS control with the value of P/N ≥ 2.1. The results were expressed as the mean (± SEM) log_10_ endpoint titers per group and the ratio of IgG2c/IgG1 in the different vaccination groups (n = 3).

### Antigen-specific cytokines secreted by splenocytes

Splenocytes (2.5 × 10^6^ cells) from three individual mice of each group were incubated in a 24-well microtiter plate for 24 h (for IL-2 detection) and 72 h (for IFN-γ and TNF-α detection) at 37°C with either RPMI1640 medium (negative control), PPD (10 μg/mL, positive control, Statens Serum Institut, Copenhagen, Denmark), or A1D4 (10 μg/mL). Supernatants from three separate wells were collected for each group and stored at -20°C until further assay. Mouse IFN-γ, TNF-α, and IL-2 ELISA kits (Multi Sciences LTD., Hangzhou, China) were employed according to the manufacturer’s instructions with the detection limits of IFN-γ, TNF-α, and IL-2 of 5 pg/mL, 8 pg/mL, and 0.22 pg/mL, respectively. The results were expressed as the mean ± SD (pg/mL) for each group (n = 3).

### Intracellular flow cytometry analysis

Splenocytes from three individual mice per group were counted and plated at 5 × 10^6^ cells/well in a 24-well plate, stimulated with A1D4 (10 μg/mL) in the presence of 1 μg/mL anti-CD28/CD49d (eBioscience, CA, USA) for 16 h, and were incubated for another 4 to 6 h after the addition of 3 μg/mL brefeldin A and 2 μM monensin solution (eBioscience). PPD (10 μg/mL) served as a positive control and cell stimulation cocktail (1 μg/mL, eBioscience) as a monitoring control. Either BSA (10 μg/mL) or RPMI1640 medium was used as a negative control. Cells were washed in FACS buffer (1% FCS-PBS), and stained for 30 min in the dark at room temperature with the following surface markers: anti-CD3-FITC (clone 17A2, eBioscience), anti-CD4-allophycocyanin-Cy7 (clone GK1.5, BD Pharmingen, CA, USA), and anti-CD8a-PE (clone53-6.7, eBioscience) mAbs. Cells were then washed again, permeabilized using the Cytofix/Cytoperm kit (BD Pharmingen) according to the manufacturer’s instructions, and stained intracellularly for 30 min using anti-IFN-PerCP-Cy5.5 (clone XMG1.2; eBioscience), anti-TNF-PE Cy7 (clone MP6-XT22; BD Pharmingen), and anti-IL-2-allophycocyanin (clone JES6-5H4; eBioscience) mAbs. Cells were subsequently washed, fixed in 1% paraformaldehyde-PBS, and then resuspended in FACS buffer. Viable lymphocytes were gated by forward and side scatter, and 1 × 10^6^ CD3^+^ events were acquired for each sample. The absolute number of PPD- or A1D4-specific CD4^+^ T and CD8^+^ T lymphocytes secreting single, double, or triple cytokines per spleen of the differently vaccinated mice was analyzed using a LSRII multicolor flow cytometer (BD Biosciences) (S1 Fig). The results were analyzed with FlowJo software (Tree Star Inc., OH, USA) and represented as the mean ± SEM per group (n = 3).

### Challenge of vaccinated mice with *M*. *tuberculosis* H37Rv

Three weeks after the last immunization, six mice in each group were challenged via the lateral vein with 1.2 × 10^6^ CFU of the virulent *M*. *tuberculosis* H37Rv strain. Four weeks later, animals were sacrificed and lungs and spleens were removed aseptically. Organs were homogenized in sterile 0.05% PBS-Tween80, and plated at 10-fold serial dilutions on Middlebrook 7H11 agar (BD, MD, USA). When required, plates were supplemented with 2-thiophenecarboxylic acid hydrazide (2 μg/mL) to selectively inhibit the possible growth of residual BCG in BCG-vaccinated mice. Plates were incubated at 37°C for 3 to 4 weeks and the protection was evaluated by assessing the bacterial load represented as mean (log_10_ CFU) ± SEM per organ for each group (n = 5).

### Lung histopathological analysis

Right lung lobes from the differently vaccinated groups were fixed in 10% formalin in PBS and embedded in paraffin. Tissue sections were prepared and stained with hematoxylin and eosin (HE) or acid-fast (AF) staining. Pathological changes were analyzed by a pathologist under a light microscope (Nikon ECLIPSE E100, Nikon, Tokyo, Japan) in a blinded fashion. The score was obtained by measuring the consolidation area of the whole field of vision (amplification 40) and calculated by the following formula: total area of consolidation/area of whole field of vision X 100%. The results were expressed as the mean of five fields of vision from each different group (n = 3).

### Statistical analysis

Nonparametric Mann-Whitney U test was used to analyze the antigen-specific IFN-γ levels in the different groups of human subjects. One-way ANOVA analysis was employed to compare the levels of antigen-specific cytokines, the numbers of polyfunctional T lymphocytes, antibody titers, and organ burdens between the different groups. A *p*-value of <0.05 was considered significant.

## Results

### Purification and identification of A1D4 polyprotein and multistage antigens

**Fig 1:**
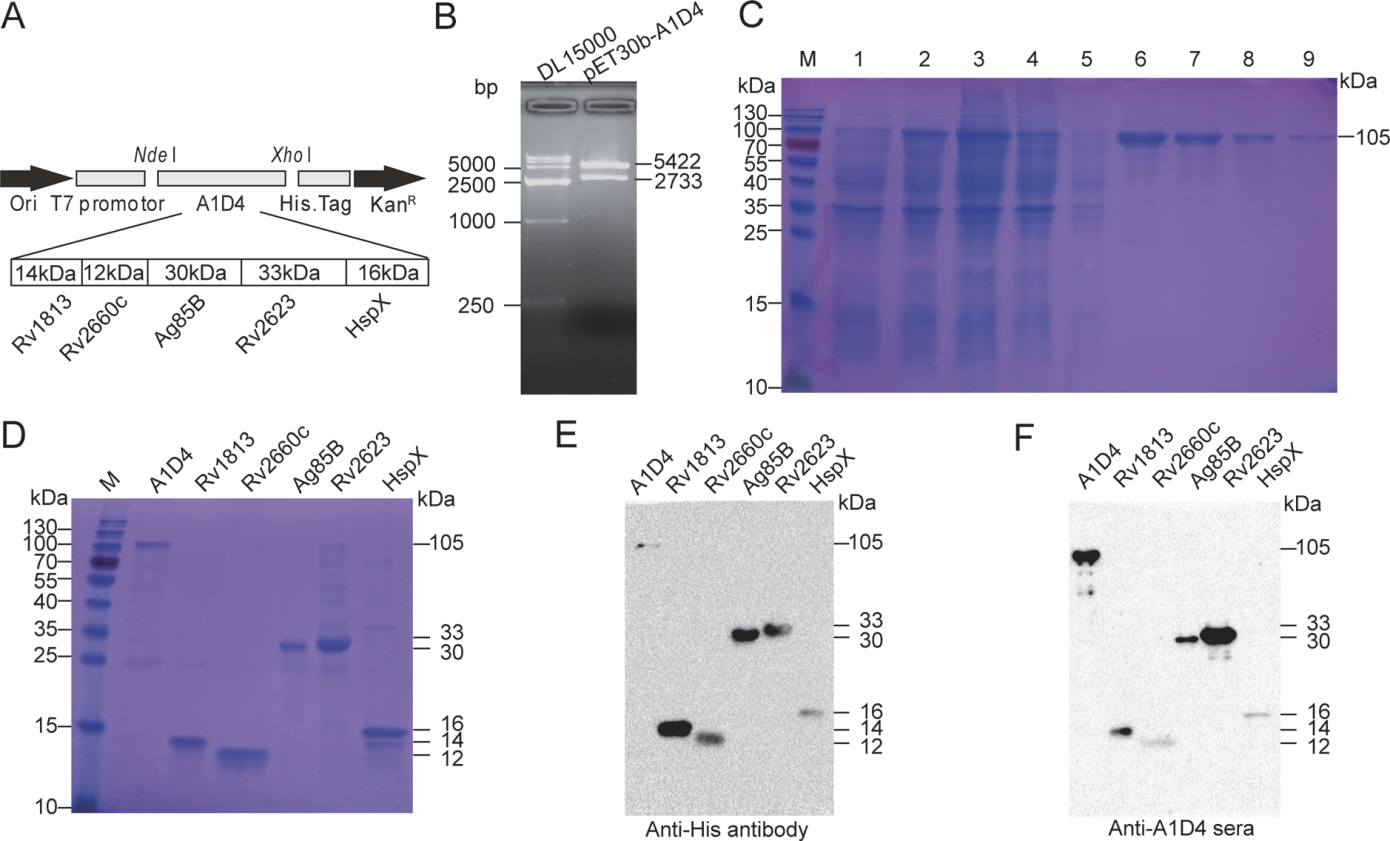
Construction, purification, and identification of A1D4 polyprotein and multistage antigens. A) Construction of the recombinant prokaryotic expression plasmid pET30b-A1D4 linking five genes of *M*. *tuberculosis*, *Rv1813*, *Rv2660c*, *Ag85B*, *Rv2623*, and *HspX* in tandem. B) Recombinant plasmid pET30b-A1D4 was characterized by restriction enzyme digestion. C) Successful expression and purification of the A1D4 polyprotein were confirmed by 15% SDS-PAGE. Lane M, protein marker (kDa); lane 1, recombinant *E*. *coli* BL21 (DE3) strain without IPTG; lane 2, recombinant *E*. *coli* BL21 (DE3) strain induced with IPTG for 4 h; lane 3, lysed *E*. *coli* pellets after sonication; lane 4, effluent after binding to the Ni-NTA column; lane 5, fraction after washing with denaturing binding buffer containing 8 M urea; lane 6–9, eluted with elution buffer containing 100 mM imidazole. D) Identification of purified A1D4 polyprotein and multistage antigens. E) Identification of purified A1D4 polyprotein and multistage antigens by western blotting with anti-His tag mcAb and F) with anti-A1D4 mouse sera.

The recombinant fusion protein A1D4 was constructed by tandem-linking the five *M*. *tuberculosis* antigens Rv1813, Rv2660c, Ag85B, Rv2623, and HspX, and expression in the plasmid pET30b-A1D4 (Fig 1), and the successful plasmid generation was verified by enzyme digestion (Fig 1). The recombinant protein A1D4 with a C-terminal His-tag was efficiently expressed in *E*. *coli* BL21 (DE3) as inclusion bodies, and purification was performed under denaturing conditions (Fig 1). The recombinant plasmid expressing *M*. *tuberculosis* antigens Rv1813, Rv2660c, Ag85B, Rv2623, and HspX, was constructed with an N- or C-terminal His-tag. SDS-PAGE analysis of all purified proteins revealed a single major band with the expected molecular weight (Fig 1). The identity of the major expected bands was confirmed by immunoblotting with an anti-His 6 mouse monoclonal antibody (Fig 1) or mouse polyclonal serum against A1D4 (Fig 1). Notably, polyclonal sera raised against A1D4 were also recognized by each of the single proteins, which confirms the presence of five antigens within the fusion polyprotein (Fig 1).

### IFN-γ profiles in response to A1D4 polyprotein and multistage antigens in human whole blood

**Fig 2:**
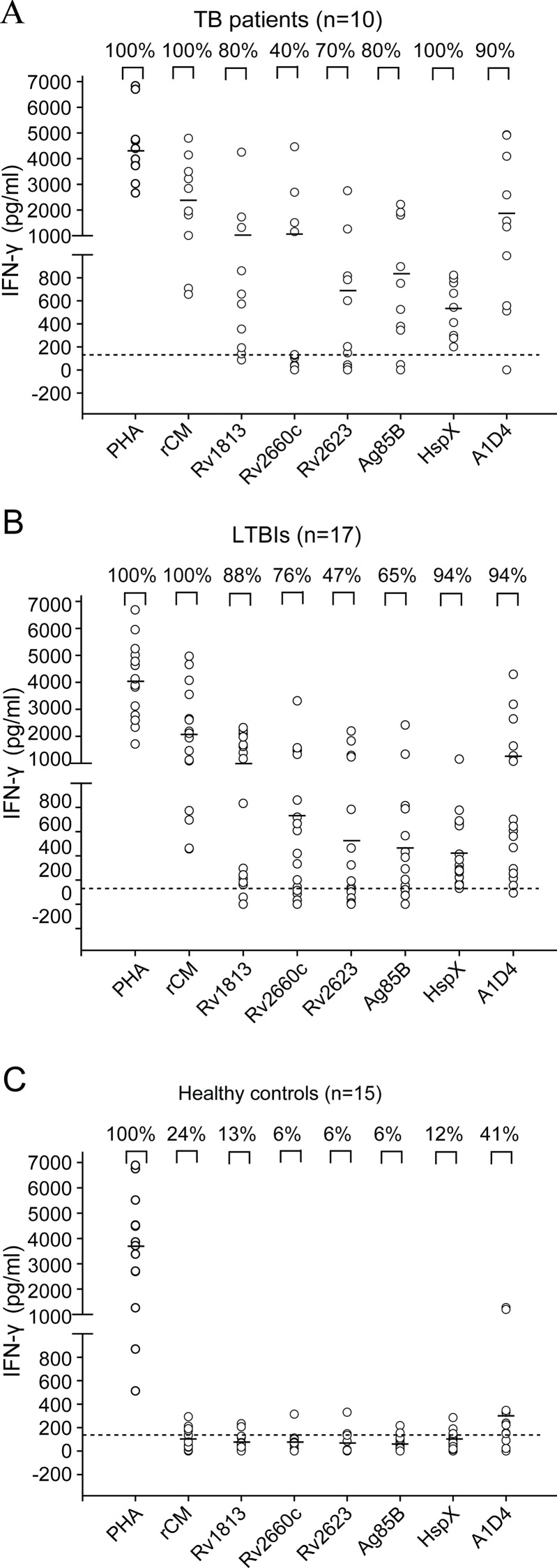
Whole blood IFN-γ profiles in response to A1D4 polyprotein and multistage antigens in active TB patients, LTBIs, and healthy controls. Whole blood samples were obtained from each subject of the active TB patient (n = 10) (A), LTBI (n = 17) (B), and healthy control (n = 15) groups (C), and stimulated with A1D4, Rv1813, Rv2660c, Rv2623, Ag85B, and HspX for 18 to 24 h. PHA was used as a positive control with a cut-off value of 100 pg/mL, and rCM (CFP21-MPT64) was used to confirm the different population characteristics based on the cut-off value of 398.5 pg/mL after clinical diagnosis. The antigen-specific concentration of IFN-γ in each sample was determined with a commercial ELISA kit with a detection limit of 4 pg/mL. Each spot represents the concentration in a sample, and median values for the different groups are shown by horizontal lines. The dotted line represents the cut-off value for a positive response to each multistage antigen, which was set arbitrarily at 3 X the mean of the negative control value of healthy controls. The nonparametric Mann-Whitney U-test was used to analyze the IFN-γ levels between the groups or different proteins. *P*<0.05 was considered statistically significant.

In order to compare the immunogenicity of the recombinant proteins, 10 active TB patients, 17 healthy LTBIs, and 15 healthy controls were selected based on the established diagnostic standard, and WBIA technology was used to evaluate the levels of IFN-γ response to purified A1D4 polyprotein and multistage antigens. As shown in Fig 2, there were no statistically significant differences between the three groups in the whole blood IFN-γ response to the positive control PHA. Interestingly, although China has a high coverage of BCG vaccination, less than 15% of healthy donors responded to antigen Ag85B and the other four latency antigens (Fig 2). Moreover, lower IFN-γ levels ranging from 0 to 390 pg/mL were induced by different multistage antigens in the healthy controls than in the TB and LTBI groups. Out of the five multistage antigens, HspX (94%) was recognized by T cells of LTBIs with the highest frequency, followed by Rv1883 and Rv2660c. T cells from all TB patients reacted easily to HspX, and more than 70% responded to Rv1883, Ag85B, and Rv2623. Notably, the five stage-specific antigens could be more easily recognized by T cells of both active TB patients and LTBIs, indicating the complex status of *M*. *tuberculosis* after infection *in vivo*. In addition, there were no statistically significant differences in the levels of IFN-γ responses to Rv1883, Rv2660c, or A1D4 in TB patients and LTBIs. Most importantly, 41% of the healthy donors, 94% of LTBIs, and 90% of TB patients responded to A1D4 (Fig 2). Our results indicate that the fusion protein A1D4 synergized the immunogenicity of the five individual antigens.

### Decreased bacterial load and lung pathological changes in A1D4-immunized mice

**Fig 3:**
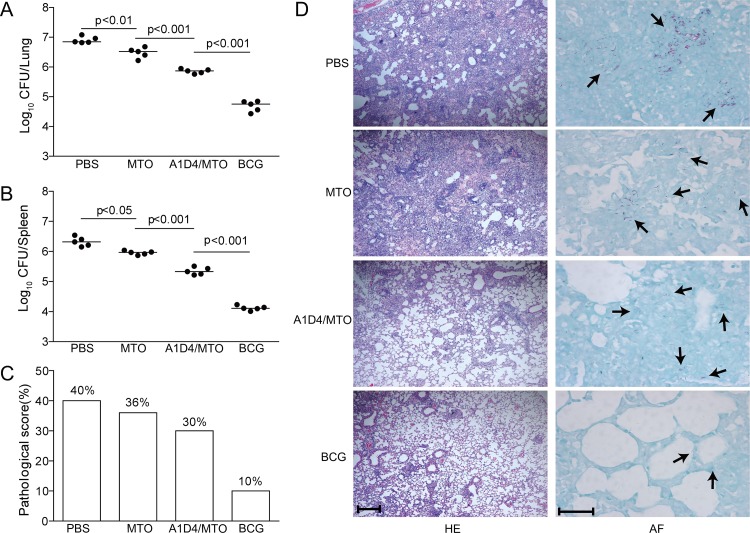
Bacterial organ load and pathological changes in the lung. Three weeks after the last immunization, C57BL/6 mice (n = 5) were challenged i.v. with 1.2 × 10^6^ CFU of the *M*. *tuberculosis* H37Rv strain. Four weeks after the challenge, spleens and lungs were harvested and the CFU numbers per organ were counted. The bacterial load in the lung (A) and spleen (B) of the different groups is represented as the mean (±SEM) log10 CFU/organ (n = 5). Lung pathological scores (C) and representative lung pathological changes from the different groups (HE, scale bar, 400 μm) are also shown (D). AF staining (scale bar, 50 μm) of lung tissue section also supported the hierarchy of lung bacterial load in the different groups (D) and arrows indicate AF-positive bacteria in the lung tissue. Challenge experiments were repeated twice with similar results.

In order to determine the protective efficacy of A1D4, vaccinated C57BL/6 mice were challenged with the live *M*. *tuberculosis* H37Rv strain. Four weeks after infection, the bacterial load in different organs and the lung pathology were assessed and compared between the groups. The highest bacterial load in the lung and spleen was obtained in the PBS control group (Fig 3) as expected. Importantly, vaccination with BCG or A1D4/MTO strongly reduced the bacterial load in both organs of C57BL/6 mice. A1D4/MTO vaccination significantly decreased the bacterial load in the lung (-1.05 log) and the spleen (-0.99 log) compared to the PBS control (*p<*0.05). In addition, although mice vaccinated with MTO also evoked a certain protection in comparison to the control group (*p<*0.05), the protection by MTO was inferior to A1D4/MTO immunization (*p<*0.05). Nevertheless, mice vaccinated with BCG experienced the strongest protective effect of all groups (*p<*0.05) (Fig 3).

Consistent with the observed varying bacterial loads in the lung, HE- and acid-fast stained lung sections showed clear differences between the groups (Fig 3). Acid-fast bacilli (AFB) were present throughout the whole lung tissue section of control mice, which exhibited the most serious lung pathology with extensive fibrosis, perivasculitis, and alveolitis (Fig 3) and also had the highest pathological score (Fig 3). Lungs from mice of the MTO group showed damage of the alveolar tissues to a lesser extent, and dispersed AFB could be detected. In contrast, lungs from mice vaccinated with A1D4/MTO featured interstitial pneumonia that was accompanied by less pronounced lung inflammation, and much fewer AFB were observed in the tissue. Only a few AFB could be detected in the alveolar tissue from BCG-vaccinated mice, which exhibited limited lung inflammation (Fig 3) and the lowest pathological score (Fig 3).

### Antibody responses induced by A1D4 in adjuvant of MTO

**Fig 4:**
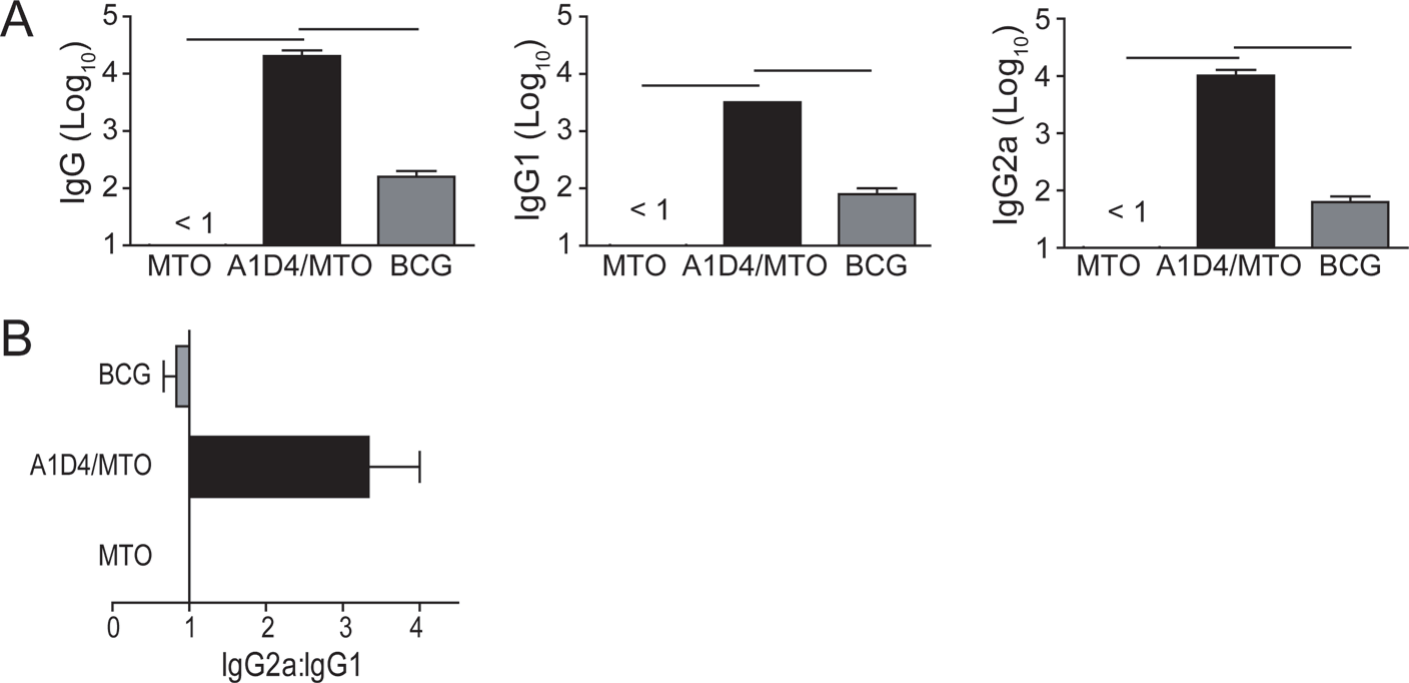
A1D4-specific IgG, IgG1, and IgG2c antibodies in immunized mice (n = 3). Three weeks after the last immunization, sera were collected from each C57BL/6 mouse of the different groups. IgG, IgG1, and IgG2c (replaced by IgG2a during the assay) antibodies against A1D4 in the immunized mice were detected by ELISA as described in detail in the Materials and Methods section. Results are shown as mean (±SEM) log_10_ endpoint titer (A) and the ratio of IgG2a:IgG1 (B) in the differently vaccinated mice (n = 3). *P*<0.05 was considered statistically significant and is indicated by the bar. This experiment was repeated twice with similar results.

The immunogenicity of A1D4 in the adjuvant of MTO was evaluated in C57BL/6 mice. A1D4-specific antibodies, including IgG, IgG1, and IgG2c, were determined by ELISA in the 9th week. As expected, there was no induction of antibodies in either the MTO alone or the PBS control group. Only mice vaccinated with A1D4/MTO induced significantly higher levels of A1D4-specific IgG, IgG1, and IgG2c antibodies (Fig 4) than the BCG group (*p*<0.05). Moreover, the ratio of IgG2c/IgG1 in the A1D4/MTO group was much higher than that of the BCG group, which indicates a switch of the IgG subclass to a Th1-based response (Fig 4).

### Antigen-specific Th1 cytokines induced by A1D4/MTO immunization

**Fig 5:**
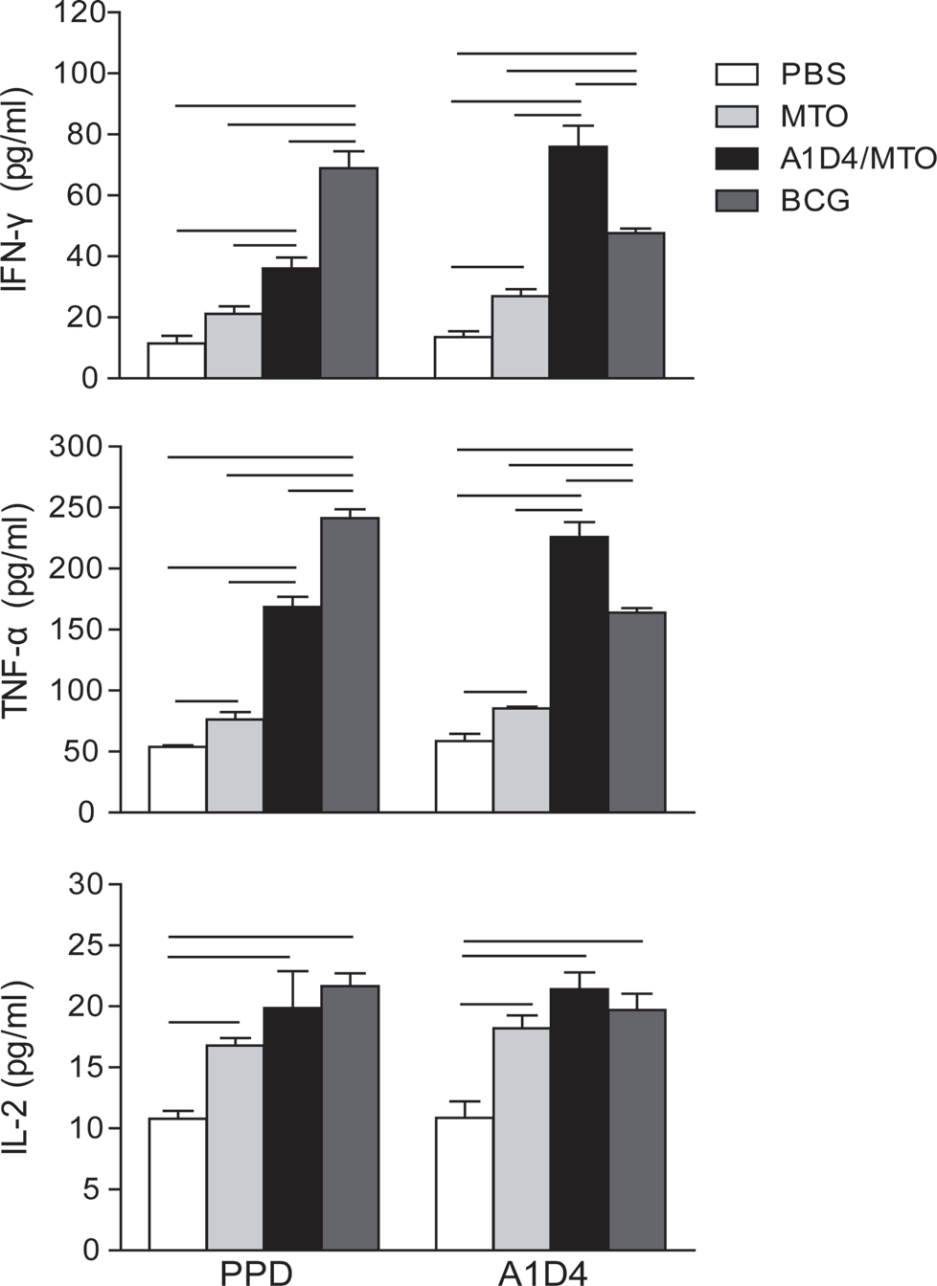
The levels of IFN-γ, IL-2, and TNF-α secreted by splenocytes of differently immunized mice. Three weeks after the last immunization, splenocytes were obtained from each mouse of the differently vaccinated groups (n = 3). 2.5 × 10^6^ cells were added into each well of 24-well microtiter plates and incubated with A1D4 protein (10 μg/mL), PPD (10 μg/mL, positive control), or complete RPMI1640 medium (negative control) for 24 h (for IL-2 detection) or 72 h (for IFN-γ and TNF-α detection) at 37°C, respectively. The cytokine concentrations in the suspension were detected with commercial ELISA kits. The results are shown as mean ± SD (pg/mL) and the bar indicates *p*<0.05. Similar results were obtained from two independent experiments.

The Th1-type response plays a fundamental role in the protection against TB [2].Th1-type cytokines, such as IFN-γ and TNF-α, which synergize to activate the microbicidal mechanism of macrophages, and IL-2, which promotes the proliferation and maturation of T cells [33], are produced in the Th1-type response. The important effects of IL-2 on the development of TB vaccine were confirmed in our [34] and other [35] previous reports. Splenocytes from the PBS control only produced very low levels of the cytokines IL-2, IFN-γ, and TNF-α, when stimulated with either PPD or A1D4 protein (Fig 5). Compared with the control, the BCG vaccine induced higher levels of IL-2, IFN-γ, and TNF-α responses to PPD (*p*<0.05) (Fig 5), which confirms the essential role of these cytokines in the BCG-induced protection against TB. Among all the groups, the highest levels of TNF-α and IFN-γ responses to PPD were induced in the BCG group, and those to A1D4 protein were conferred in the A1D4/MTO group (*p*<0.001). In addition, there were no statistically significant differences in the levels of IL-2 responses to PPD or A1D4 between the A1D4/MTO, BCG, and MTO groups. Taken together with previous results, our study suggests that the protection conferred by A1D4/MTO is also largely dependent on A1D4-specific Th1-type responses.

### Polyfunctional T cells in response to PPD and A1D4

**Fig 6:**
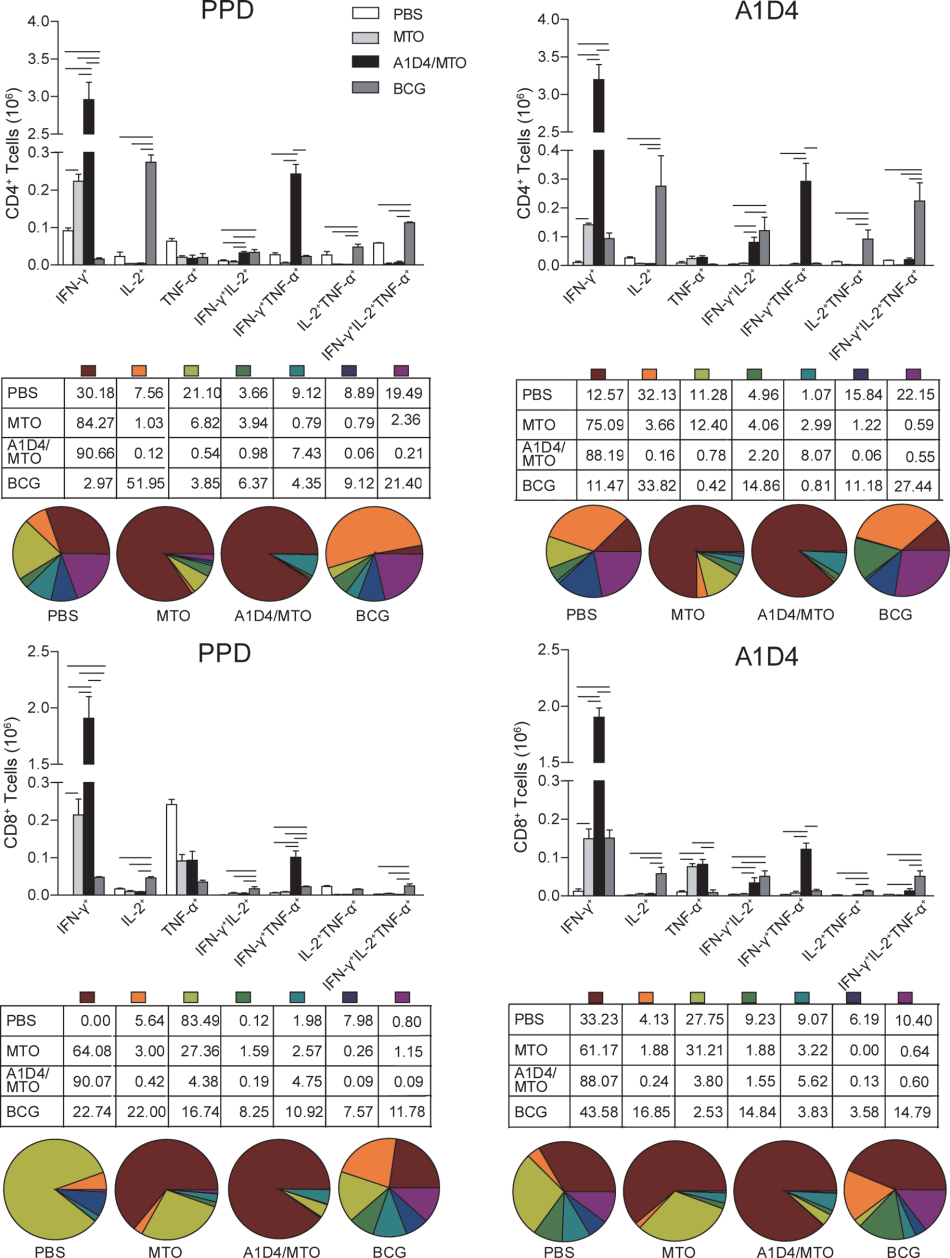
Polyfunctional expression of IFN-γ, TNF-α, and IL-2 by CD4+ or CD8+ T cells in response to PPD or A1D4 analyzed by multicolor flow cytometry. Splenocytes of each mouse from the different groups were collected and counted. 5 × 10^6^ cells/well in a 24-well plate were stimulated with A1D4 (10 μg/mL) or PPD (10 μg/mL) as described in detail in the Materials and Methods section. Intracellular cytokine profiles for IFN-γ, TNF-α, and IL-2 in individual cells were measured by multicolor flow cytometry by gating for CD4^+^ or CD8^+^ T cells. Every possible combination of cytokines is shown on the x-axis of the bar chart and the absolute number of antigen-specific T cells expressing any combination of cytokines is shown as mean ±SEM in the different groups (n = 3). Similar results were obtained from two independent experiments (the bar indicates *p*<0.05).

In order to obtain protective biomarkers for clinical or experimental evaluation of TB vaccine candidates, seven combinations of three cytokines in CD4^+^ or CD8^+^ T cells were compared between the different groups. Relative to the control, the BCG vaccine only significantly increased the absolute number of PPD-specific single IL-2^+^ CD4^+^ T cells and IL-2^+^IFN-γ^+^TNF-α^+^, IL-2^+^IFN-γ^+^, and IL-2^+^TNF-α^+^ CD4^+^ T cells (*p<*0.05). More than half of the seven combinations of CD4^+^ T cell responses to PPD or A1D4 in BCG-vaccinated mice belonged to single IL-2^+^ and IFN-γ^+^IL-2^+^TNF-α^+^ cells (Fig 6), which could be the key factors correlating with BCG-induced protection. Among these four combinations, only PPD-specific IL-2^+^IFN-γ^+^ CD4^+^ T cells were more significantly increased in the A1D4/MTO group (only 0.98% of the seven combinations in CD4^+^ T cells) (*p<*0.05), and especially increased to 2.21% in response to A1D4 (*p<*0.05) compared with the PBS and MTO controls (Fig 6). In contrast, A1D4/MTO immunization mainly increased PPD- or A1D4-specific single IFN-γ^+^ CD4^+^ T cells and the following IFN-γ^+^TNF-α^+^ CD4^+^ T cells (*p<*0.05). On the other hand, the BCG vaccine evoked mainly PPD-specific single IFN-γ^+^ and single IL-2^+^ CD8^+^ T cells, and the following IL-2^+^IFN-γ^+^TNF-α^+^, IFN-γ^+^TNF-α^+^, and IL-2^+^IFN-γ^+^ CD8^+^ T cells, when compared with control mice (*p<*0.05). Among these five combinations in CD8^+^ T cells, the highest absolute number of single IFN-γ^+^ and IFN-γ^+^TNF-α^+^ CD8^+^ T cells response to either PPD or A1D4 protein were obtained only in A1D4/MTO-vaccinated mice (*p<*0.05) (Fig 6) followed by A1D4-specific IL-2^+^IFN-γ^+^ and IL-2^+^IFN-γ^+^TNF-α^+^ CD8^+^ T cells, which also increased more significantly in the A1D4/MTO group compared to the PBS control (*p<*0.05).

## Discussion

Due to the serious threat of a TB epidemic to public health [36] and the lack of protection by the current BCG vaccine, protein subunit vaccines present a compelling area of research for improved control of TB. Nevertheless, it is very difficult to screen promising antigens encoded by the genome of *M*. *tuberculosis* that contains 4018 ORFs [37]. The approach of combining clinical diagnosis with WBIA has been presented in our previous studies [38, 39]. This method was reconfirmed as a reasonable, simple, and effective way to screen target antigens with vaccine potential, in comparison with other methods such as biochemical separation [3, 4], comparative [40] or immune proteomics [41], comparative genomics [42], transcriptomics [43], or even direct animal challenge experiments [21]. In this study, five multistage antigens were selected based on the interaction between *M*. *tuberculosis* and its host, and were confirmed to be recognized by T cells of human TB subjects. The polyprotein A1D4 consisting of five multistage antigens was constructed and its immunogenicity and protective efficacy in adjuvant of MTO was evaluated in vaccinated C57BL/6 mice. Our results demonstrated that the subunit vaccine A1D4/MTO could provide more significant protection against *M*. *tuberculosis* infection than the PBS control and adjuvant MTO alone, as evidenced by lower bacterial load in the lung and spleen, less pathological changes in the lung, and more significantly increased A1D4-specific Th1-type responses.

The BCG vaccine has been used as the gold standard to evaluate the efficacy of vaccine candidates in TB animal models. Nonetheless, it has proven difficult to find a new subunit vaccine that provides stronger protective efficacy than BCG, because BCG is an attenuated strain derived from *M*. *bovis* and secretes at least 800 proteins [44]. Naturally, BCG infects APCs including macrophages and DCs as a way of presenting antigens after immunization [45]. Although protection by A1D4/MTO is not as good as BCG, differential immunogenicity and protective efficacy observed in the BCG- and A1D4/MTO-immunized mice suggest that this is a good model for finding effective biomarkers associated with protection, which would help assess vaccine-induced protection and develop the next generation of vaccines. The occurrence and progression of TB infection is mainly associated with cell-mediated immunity of the host. BCG is a stronger inducer of CD4^+^ Th1 cells than CD8^+^ T cells, partially because BCG lacks the ESX-1 mechanism [46] and is unable to translocate from the phagolysosome to the cytosol. Antigens localized to the phagosome would therefore be processed and presented mainly via MHC II molecules. Identifying and understanding effective biomarkers with protection capacity is of extreme significance, because the BCG vaccine provides only limited protection against adult TB, and further enhanced Th1 response boosted by MVA85A [47] fails to provide enhanced efficacy in vaccinated infants [48].

Polyfunctional analysis was first reported in HBV vaccine-induced T cell responses [49] and was highlighted in a few recent clinical trials of TB vaccine candidates [50, 51] and in a mouse model using *Leishmania* [52]. Although polyfunctional T cells may be involved in the protection against TB, effective biomarkers of polyfunctional T cells with vaccine-induced protection remain to be defined. It is worth noting that polyfunctional T cell responses to PPD or A1D4 in BCG- and A1D4/MTO-vaccinated mice were quite different. Our results clearly demonstrate the phenotypic and functional heterogeneity of antigen-specific CD4^+^ T cells and CD8^+^ T cells induced by BCG or A1D4/MTO. In particular, the number of single IL-2^+^ and IFN-γ^+^IL-2^+^TNF-α^+^ CD4^+^ T cells was significantly increased in BCG-vaccinated mice. Although the highest absolute number of single IFN-γ^+^ CD4^+^ T cell responses to PPD or A1D4 was conferred by A1D4/MTO-immunized mice, it did not positively correlate with the strongest protection against infection among all the groups, which supports the previous conclusion [53]. In addition, H56 in adjuvant of CAF01-vaccinated mice also significantly evoked IL-2^+^IFN-γ^+^TNF-α^+^- and IL-2^+^ IFN-γ^+^-secreting CD4^+^ T cells and provided a protection against *M*. *tuberculosis* infection comparative with BCG [19], while mainly the number of IFN-γ^+^TNF-α^+^ CD4^+^ T cells increased in ID93/GLA-SE-immunized mice with protection inferior to BCG [29]. The same observation was demonstrated in A1D4/MTO-vaccinated mice, which also significantly elicited PPD- and A1D4-specific single IFN-γ^+^- and IFN-γ^+^TNF-α^+^- secreting CD4^+^ T cells in this study. Single IFN-γ^+^ CD4^+^ T cells are associated with viral primary infection with high viral load, while chronic viral infection is related to single IL-2^+^ or IL-2^+^IFN-γ^+^ CD4^+^ T cell responses [54]. Polyfunctional analysis in HIV and *M*. *tuberculosis* co-infections revealed that HIV viral load positively correlated with single IFN-γ^+^ CD4^+^ T cells yet negatively correlated with *M*. *tuberculosis*-specific IL-2^+^-secreting CD4^+^ T cells [54]. Antiretroviral therapy promotes significant increases of IL-2^+^IFN-γ^+^- and IL-2^+^TNF-α^+^- secreting CD4^+^ T cells and hinders the progression from infection to active TB, while single IFN-γ^+^ CD4^+^ T cells declined significantly [55]. In addition, IL-2^+^IFN-γ^+^TNF-α^+^-secreting CD4^+^ T cells in response to antigen Ag85B or HspX were also more easily detected in active TB patients than LTBI subjects [56,57]. Compared with pretreatment, there was a clear increase in the proportions of IL-2^+^IFN-γ^+^ secreting CD4^+^ T cells following treatment, which is similar to the pattern observed in the LTBI population [56]. All of these findings indicate that antigen-specific single IL-2-secreting cells and different combinations with IL-2-secreting CD4^+^ T cells are positively correlated with vaccine-induced protection against TB. Therefore, the increased number of IL-2^+^IFN-γ^+^ CD4^+^ T cells should be closely related to the protection in A1D4/MTO-vaccinated mice, whereas the absence of single IL-2^+^, IFN-γ^+^IL-2^+^TNF-α^+^, and IL-2^+^TNF-α^+^ CD4^+^ T cells might explain the reason for a protection by A1D4/MTO inferior to BCG.

CD8^+^ T cells were confirmed to play an important role in the protection in mice or non-human models, which might take part in the process of preventing the reactivation of LTBI and compensate the role of CD4^+^ Th1 response against primary infection [58]. The optimal protection induced by vaccines may be obtained by combination of CD4^+^ Th1 and CD8^+^ T cell responses. However, the role of polyfunctional CD8^+^ T cells in the vaccine induced-protection against TB infection remains unclear. In this study, the frequency of single IFN-γ^+^ CD8^+^ T cells was increased more significantly in A1D4/MTO- than BCG-vaccinated mice. Previous studies have confirmed the protective role of IFN-γ-secreting CD8^+^ T cells in a mouse TB model [59] and in humans [60]. In addition, much more PPD- or A1D4-specific IFN-γ^+^TNF-α^+^ CD8^+^ T cells were induced in A1D4/MTO-vaccinated mice than in those vaccinated with BCG. IFN-γ^+^TNF-α^+^ CD8^+^ T cells in response to latency antigens including HspX was the most frequently identified in long-term latently infected individuals that did not progress to active TB [61]. Therefore, these CD8^+^ T cell types might present the advantages of A1D4/MTO over BCG to protect against latent infection, providing indirect evidence to support the popular strategy of BCG priming and heterologous boosting with A1D4/MTO in future studies in order to remedy CD8^+^ T-cell responses after BCG vaccination and limit infection and progression to active TB.

## Conclusions

The observations of the present study suggest that vaccination with A1D4/MTO, consisting of four antigens related to the phase of latency and one secreted antigen related to the phase of primary infection, could provide effective protection against *M*. *tuberculosis* infection in vaccinated mice and present an effective strategy for the development of the next generation of vaccines. Our results warrant further evaluation of A1D4/MTO in different animal models, including latent infection and BCG vaccination boosting.

## Supporting Information

S1 Fig Representative flow cytometry showing the gating strategy with the FlowJo 7.61 software for the identification of single and polyfunctional CD4^+^ and CD8^+^ T cells per spleen from differently vaccinated mice. (TIF)

S1 Table Primers and thermal cycle parameters for cloning of *M*. *tuberculosis* antigens. (XLS)

S2 Table Characteristics of human subjects. (XLS)
