# Supplementary figures and images for: Protection against Mycobacterium tuberculosis Infection Offered by a New Multistage Subunit Vaccine Correlates with Increased Number of IFN-γ+IL-2+ CD4+ and IFN-γ+ CD8+ T Cells
Source: PLoS One. 2015 Mar 30;10(3):e0122560. doi: 10.1371/journal.pone.0122560 (PMC4378938; doi:10.1371/journal.pone.0122560)

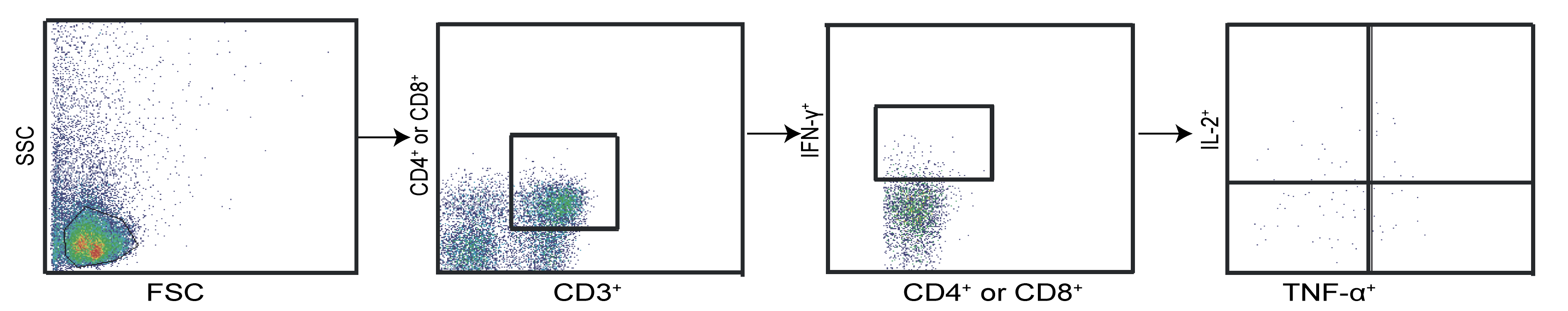

Supplement: S1 Fig — (TIF) [file pone.0122560.s001.tif]
